# Gastrointestinal toxic effects in patients with cancer receiving platinum-based therapy

**DOI:** 10.7150/jca.37777

**Published:** 2020-03-04

**Authors:** Hamzah Abu-Sbeih, Niharika Mallepally, Ryan Goldstein, Ellie Chen, Tenglong Tang, Uzoamaka K. Dike, Mazen Al-Asadi, Shannon Westin, Daniel Halperin, Yinghong Wang

**Affiliations:** 1 Department of Gastroenterology, Hepatology, and Nutrition, The University of Texas MD Anderson Cancer Center, 1515 Holcombe Blvd, Houston, TX 77030, USA; 2 Department of Internal Medicine, Baylor College of Medicine, 6620 Main St, Houston, TX 77030, USA; 3 Department of Internal Medicine, McGovern Medical School at The University of Texas Health Science Center at Houston, 6431 Fannin St, Houston, TX 77030, USA; 4 Department of Gynecologic Oncology and Reproductive Medicine, The University of Texas MD Anderson Cancer Center, 1515 Holcombe Blvd, Houston, TX 77030, USA; 5 Department of Gastrointestinal Medical Oncology, The University of Texas MD Anderson Cancer Center, 1515 Holcombe Blvd, Houston, TX 77030, USA

**Keywords:** platinum, carboplatin, cisplatin, gastrointestinal toxicity, colitis

## Abstract

**Background**: Platinum-based therapy (PBT) can be limited by gastrointestinal adverse events, particularly PBT-related colitis and diarrhea (PCD). We studied clinical features, treatments, and outcomes of PCD.

**Methods**: This was a retrospective study of cancer patients who received PBT and colonoscopic evaluation for PCD symptoms from 2009 to 2018.

**Results**: Of 36,595 patients who received PBT, 86 (0.2%) met inclusion criteria. Median time from PBT initiation to PCD was 66 days. Regarding PBT type, 47% of the patients received carboplatin, 31% cisplatin, and 22% oxaliplatin. Median duration of PCD symptoms was 20 days. Colonoscopy revealed mucosal ulceration in 34% of the patients and nonulcerative inflammation in 33%. Half of the cohort needed hospitalization for PCD (49%). The majority received treatment for PCD (59%): immunosuppressive therapy in 21%, antibiotics in 27%, antimotility agents in 22%, and intravenous fluids in 51%. Eight patients (9%) were admitted to the intensive care unit for PCD management. Six patients (7%) experienced colonic perforation that required surgical intervention; two of them had gastrointestinal tumors. Physicians restarted PBT in 37 (43%) patients; 8 (22%) of them had PCD recurrence that was managed expectantly. Colonic perforation occurred more frequently with use of oxaliplatin and cisplatin than carboplatin (*P*=0.05). The median duration of PCD symptoms was longer in patients receiving carboplatin or cisplatin than in those receiving oxaliplatin (*P*=0.182).

**Conclusions**: PCD is rare, but in a small subset of patients, it can lead to serious complications. Treatment of PCD is mainly supportive, but immunosuppressive therapy may be required.

## Introduction

Platinum-based therapy (PBT) includes that with carboplatin, cisplatin, or oxaliplatin and is used for a wide variety of malignancies, including ovarian, lung, head and neck, and rectal cancers; neuroblastoma; and many others 1. Because these agents have been in use since the early 1980s, they have a well-established efficacy profile and are on the World Health Organization's list of essential medicines 2. Structurally, carboplatin and cisplatin are analogs of one another and have similar active metabolites. Functionally, these three agents have slightly different mechanisms of action. Platinum-based antineoplastic agents serve as DNA adducts that cause DNA proteins to cross-link. This cross-linking inhibits further DNA synthesis and repair in malignant cells 1, 3.

As reported in the literature, platinum agents are mainstays in lung cancer chemotherapy, often as first-line treatment. Studies have also supported the use of PBT for ovarian, cervical, uterine, and colorectal cancer 4-8. In short, the applications of PBT are broad and well documented. Nevertheless, the utility of PBT has been impacted by its side effects. Platinum agents have dozens of reported side effects, some of which differ according to the agent. Specifically, oxaliplatin can be neurotoxic, carboplatin can cause myelosuppression, and cisplatin can be both nephrotoxic and neurotoxic. Use of all three agents has been associated with bone marrow toxicity, hepatotoxicity, mucositis, and, most notably, abdominal complaints. A review of treatment with cisplatin documented its ability to create electrolyte imbalances 9. For currently used platinum agents, side effects are often dose-dependent and require monitoring using serial laboratory tests.

The literature on the physiology, efficacy, and general adverse effects of PBT is comprehensive. However, virtually no studies have focused on colitis related to use of these agents. Given that PBT can last for months and is given to potentially debilitated cancer patients, understanding PBT-related colitis and diarrhea (PCD) is essential. Therefore, we performed the study described herein to identify and characterize platinum-related colitis.

## Methods

### Patient Selection and Data Collection

After obtaining approval from the Institutional Review Board at The University of Texas MD Anderson Cancer Center, a retrospective, descriptive, single-center study of cancer patients who underwent PBT was conducted. Specifically, patients who received PBT with oxaliplatin, carboplatin, or cisplatin and underwent endoscopy along with biopsy analysis from 2009 to 2018 were screened. Patients in whom colitis symptoms developed following PBT and who underwent colonoscopic evaluation with biopsy analysis for PCD were included. PBT could have been given in a single-agent regimen or as part of multiagent one, as neoadjuvant or adjuvant treatment, and under a clinical trial or otherwise. Patients with competing etiologies of colitis, such as graft-versus-host disease and infectious colitis, were excluded.

### Clinical Characteristics

After the study cohort was identified, their data were extracted from institutional electronic medical records. The data included demographics (age, sex, and race/ethnicity), medical and oncological history, medical comorbidities included in the Charlson Comorbidity Index,^10^ smoking status, and recent nonsteroidal anti-inflammatory drug or aspirin use (within the previous 3 months). Oncological history was screened for variables related to 1) cancer type, 2) chemotherapy regimens (agents were listed individually), 3) noncolitis adverse events, 4) resumption of PBT after resolution of colitis, and 5) radiation therapy delivered to the abdominal region.

### Clinical Presentation and Treatment of PCD

Clinical symptoms and signs of PCD in the study patients were diarrhea, abdominal pain, nausea and vomiting, blood or mucous in stool, and concurrent fever. At the time of PCD onset, findings on computed tomography (CT) scans suggestive of colitis included mucosal enhancement, colonic wall thickening, and pericolonic fat stranding. The presence of bacteremia or a gastrointestinal infection at the time of PCD development was documented. Of note, treatments of PCD symptoms were recorded qualitatively and included supportive care, antimotility agents, pain medications, intravenous fluids, antibiotics, immunosuppressive therapy, and surgery.

### Endoscopic and Histological Evaluation

The patients' colonoscopy and sigmoidoscopy data were also retrieved. Examined variables included features of ulcers, the presence of erosion, the patterns and locations of inflammation (e.g., erythema, friability, nodularity, loss of vascular pattern, edema), and the presence of exudates. Histological records were examined for signs of acute inflammation, such as neutrophilic infiltrates, cryptitis, crypt abscesses, apoptosis, and eosinophilia. Chronic inflammation patterns were also noted, including lymphocytic infiltrates in the lamina propria, crypt architectural distortion, Paneth cell metaplasia, and granuloma or generalized chronic active inflammatory patterns. Microscopic colitis features with lymphocytic or collagenous patterns in the epithelium were also noted.

### Clinical Outcome

Clinical outcomes of PCD examined in the patients' charts included need for admission to the hospital or intensive care unit (ICU); duration of hospitalization, gastrointestinal symptoms, and PCD treatment; PCD-related colonic perforation; recurrence of gastrointestinal symptoms after improvement; repeated endoscopic evaluation; and death.

### Statistical Analysis

Statistical analyses were carried out using the SPSS software program (version 24.0; IBM Corporation, Armonk, NY). Categorical variables were summarized using frequencies and proportions. Continuous variables were presented as medians and interquartile ranges (IQRs) or means (± standard deviations). The Fisher exact and chi-square tests were used to compare categorical variables. The Wilcoxon rank sum test was used to compare continuous variables. All statistical tests were two-sided. *P* values of up to 0.05 were considered statistically significant.

## Results

### Patient Characteristics

**Figure 1:**
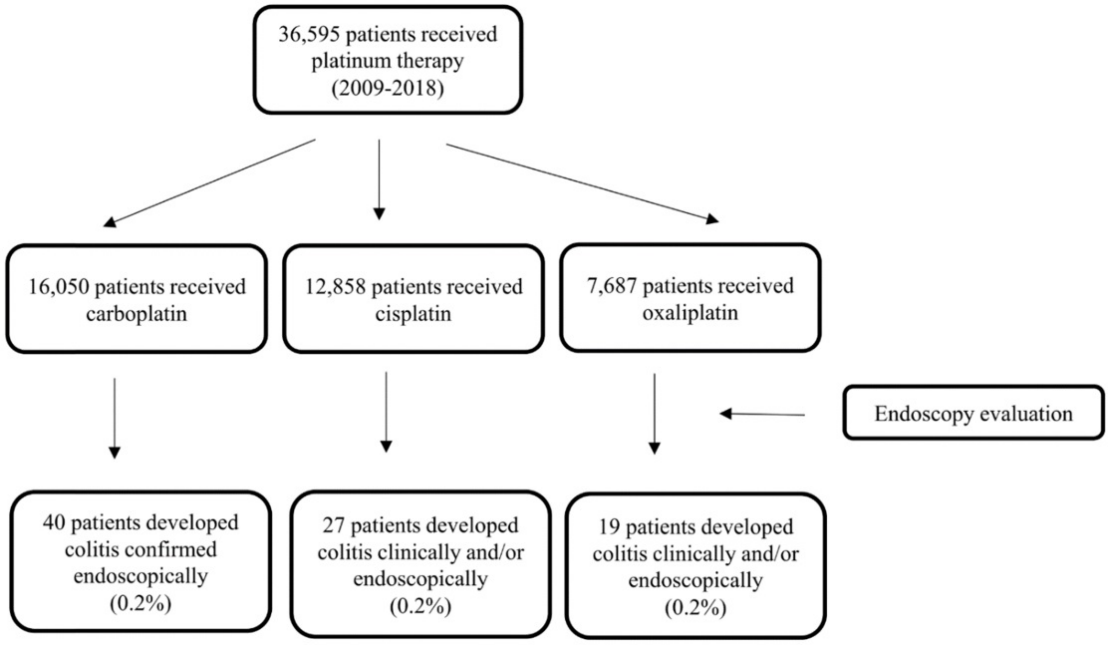
Flowchart of the study patients.

Of 36,595 patients who received PBT from 2009 to 2018, 16,050 received carboplatin, 12,959 received cisplatin, and 7,687 received oxaliplatin. A total of 86 (0.2%) patients had PCD confirmed via endoscopy (Fig. 1), thus meeting the inclusion criteria for our study. Their median age was 57 years (IQR, 49-66 years). The population's clinical characteristics are outlined in Table 1. Only 11% of them had pre- existing inflammatory bowel disease (IBD), among them, 4 had active disease activity requiring medical treatments. The most common primary malignancy was gastrointestinal tract cancer (28%) followed by lung (21%), gynecological (19%), and genitourinary (11%) cancers. Almost half of the study participants received carboplatin (47%), with 31% receiving cisplatin and 22% receiving oxaliplatin. The median duration of PBT was 51 days (IQR, 21-114 days).

### Clinical Presentation and PCD Treatment

PCD symptoms developed in the participants at a median of 66 days (IQR, 9-153 days) after starting PBT. During initial presentation, the most common clinical symptom was diarrhea (62%). Abdominal pain and blood or mucous in stool were also common, occurring in 48% and 43% of patients, respectively. Symptoms lasted a median of 20 days (IQR, 7-56 days). PCD led to PBT discontinuation in 17% of the patients. Moreover, PBT was restarted after resolution of PCD in 43% of the patients. Only 9% of the participants had recurrent gastrointestinal symptoms.

At the time of PCD onset, the patients' median absolute neutrophil count was 3.1 k/mm^3^ (IQR, 1.4-4.2 k/mm^3^). Forty patients underwent CT, 64% of whom had abnormal findings. Seven patients had confirmed infections (Clostridium difficile infection in 6 and stool staphylococcus aureus infection in 1). Most patients (59%) received treatment of PCD symptoms, which included antibiotics (27%), immunosuppressants (21%), antimotility agents (22%), and, most frequently, intravenous fluids (51%). The median length of PCD treatment was 14 days (IQR, 7-29 days).

The difference in the time from PBT to symptom onset according to platinum drug was not significant (*P*=0.428) (Table 2). Patients receiving oxaliplatin developed diarrhea less frequently than those receiving other PBT (P=0.02) (*P*=0.002). Also, the incidence of blood or mucous in stool (*P*=0.090) and fever (*P*=0.051) tended to be lower in patients receiving oxaliplatin than in those receiving the other two agents. Abdominal pain and nausea and vomiting were equally prevalent in patients receiving oxaliplatin, carboplatin, and cisplatin. The median duration of PCD symptoms was 7 days in patients who received oxaliplatin, whereas it was 30 days in those who received carboplatin and 25 days in patients who received cisplatin (*P*=0.182).

Participants who presented with diarrhea (*P*<0.001), vomiting (*P*<0.006), or abdominal pain (*P*<0.001) were more likely to undergo treatment of PCD symptoms than were those who did not receive treatment (Table 3). Those who presented with fever (*P*=0.081) or blood or mucous in stool (*P*=0.077) were only somewhat more likely to receive treatment. Recurrent PCD was equally present in the treatment and nontreatment groups. The duration of PCD symptoms was similar in these two groups (*P*=0.247). The use of different treatment regimens, including immunosuppressants, intravenous fluids, antibiotics, and antimotility agents, was equally prevalent in participants who received oxaliplatin, carboplatin, and cisplatin.

### Endoscopy and Histology

**Figure 2:**
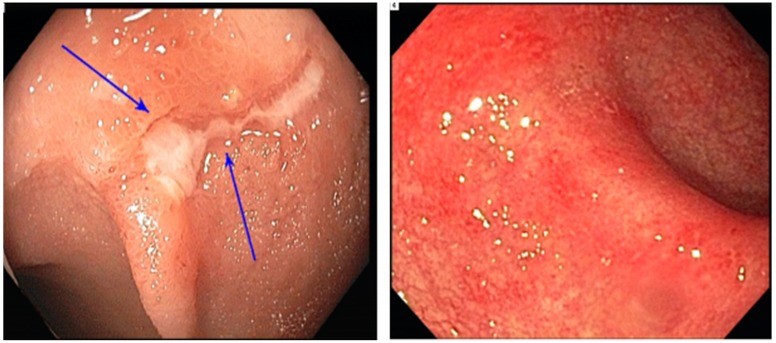
Endoscopy presentation of platinum-related colitis. Superficial ulcers (left); and diffuse erythema and edema (right).

Colonoscopic findings were roughly evenly distributed: normal colonic tissue in 34% of the participants, mucosal ulceration in 34%, and nonulcerative inflammation in 33% (Fig. 2). Regarding the pattern of endoscopic inflammation, 40% of colonoscopies demonstrated patchy inflammation, whereas 23% demonstrated diffuse inflammation. Thirty-three percent of abnormal endoscopic findings were in the left colon only, and 7% were in the right colon only. Only 26% of the study participants had involvement of the entire colon (Table 1).

In terms of ulcers, nonulcerative inflammation, and normal colonic tissue, we observed no significant endoscopic differences regarding these features between the treatment and nontreatment groups. Also, the platinum drug received did not correlate with endoscopic findings. However, diffuse inflammation was most common in carboplatin recipients (50%) and least common in oxaliplatin recipients (11%). The trend for patchy inflammation was reversed, as it was most common in oxaliplatin recipients (89%) and least common in carboplatin recipients (50%). Of note, this association was only partially significant and appeared to be secondary to the varying presence of inflammation in the oxaliplatin group (*P*=0.098) (Tables 2 and 3).

Histologically, inflammation was categorized as active or chronic. Seventy-six percent of the participants had histologies demonstrating active inflammation, whereas 55% had chronic inflammation. We found active and chronic inflammation patterns equally in the treatment and nontreatment groups. The presence of active or chronic inflammation did not differ according to type of PBT (Table 3).

### Clinical Outcomes

Forty-nine percent of the patients had to be hospitalized, 9% needed ICU unit admission, and 7% had a colonic perforation requiring surgery. Three of these 6 patients with complications received bevacizumab as part of adjuvant chemotherapy. Complications were less likely in those who received carboplatin than in those given the other two platinum drugs (*P*=0.05). Recurrent gastrointestinal symptoms occurred in 25% of patients who resumed PBT after PCD onset (*n*=37). Recurrent PCD was absent from oxaliplatin recipients and most common in carboplatin recipients (18%; *P*=0.046).

Restarting PBT after the occurrence of PCD was most common in the carboplatin group (58%) followed by the cisplatin (33%) and oxaliplatin (26%) groups (*P*=0.037). Only 16% of the participants underwent repeat endoscopy. We noted endoscopic remission in 36% of the study participants and histological remission in 25% of them. Furthermore, the treatment and nontreatment groups were equally likely to receive PBT after occurrence of PCD or to undergo repeat endoscopy. Repeat endoscopy was most common in the oxaliplatin group and least common in the cisplatin group (*P*=0.02).

## Discussion

This retrospective study sheds light on the importance of the clinical, endoscopic, and histological characteristics of PCD and its outcomes. The incidence of PCD after PBT was very low, but PCD can lead to significant morbidity, such as colonic perforation. Treatment with carboplatin or cisplatin was more likely to cause diarrhea and blood or mucous in stool than was treatment with oxaliplatin. Although information regarding PCD in the literature is scarce, a significant proportion of our cohort received various types of nonspecific PCD treatment.

Authors have reported that the incidence rate for diarrhea and abdominal pain associated with PBT ranges from 30% to 50% in patients.^11^ However, the incidence of endoscopy-confirmed colitis has yet to be reported. In our cohort, the incidence of PCD was very low, as we included only patients who underwent colonoscopic evaluation. Several case reports and studies demonstrated gastrointestinal side effects of PBT, especially that with carboplatin and cisplatin.^12^-^17^ They described PCD as a form of ischemic colitis, IBD, infectious colitis, or neutropenic enterocolitis given the overlapping presentations. Therefore, to account for possible confounding factors, we included only patients who underwent colonoscopy and biopsy. In our cohort, 4 out of 9 patients had active status of IBD at the time of PBT. Confounding factors from active IBD symptoms or exacerbation with PCD need to be considered. In addition, despite that the average absolute neutrophil count in the patients was in the normal range (perhaps because symptom onset occurred at a median of 66 days after PBT began), frequently experienced neutropenic episodes expected during PBT treatment could still have had occurred, affecting colitis presentation. With these differential diagnoses for colitis, endoscopy and histology evaluation will provide valuable information to clarify the etiology of colitis and guide subsequent management.

Our findings bring up the question of whether to treat PCD upon initial presentation. Given that a minority of patients could have bacteremia or a gastrointestinal infection, we must determine whether antibiotics should be given immediately in cases of suspected colitis. Standard of practice dictates that when a cancer patient presents with nonspecific abdominal or constitutional symptoms, broad- spectrum antibiotics are given. However, indiscriminate use of antibiotics can lead to an increase in the incidence of gastrointestinal symptoms. Some PCD patients are not immunocompromised or febrile, and so their PCD is best managed via expectant care (i.e., intravenous fluids, antimotility agents).

Analysis of patient characteristics demonstrated that in the 49% of participants who needed hospitalization, the median duration of hospitalization ranged from 5 to 9 days. Of note, PCD symptoms lasted an average of 20 days, indicating that they were mild enough to not always necessitate hospitalization. This finding also demonstrates that treatment of colitis symptoms, which was primarily conservative, could have continued in the outpatient setting. Unsurprisingly, PCD symptoms were manageable enough that PBT had to be stopped in only 17% of cases.

With all three platinum drugs, we found that patients with PCD presented with nonspecific abdominal pain and nausea and vomiting. In light of the fact that 28% of the patients had primary gastrointestinal malignancies, such nonspecific symptoms could be attributed to multiple causes. However, diarrhea and blood or mucous in stool were equally common in the carboplatin and cisplatin groups but rare in the oxaliplatin group. Therefore, when evaluating gastrointestinal symptoms in patients with a history of PBT exposure, an important step is to note that 1) oxaliplatin recipients present with mild, nonspecific symptoms and 2) symptoms may not be attributed to malignancy, infection, or graft-versus-host disease.

Unsurprisingly, the presence of PCD symptoms was significantly associated with treatment of these symptoms. However, participants with longer symptom durations were not more likely to receive PCD treatment. This demonstrates that PCD symptoms were likely self-limited. However, it may also demonstrate that some patients have baseline intermittent gastrointestinal symptoms (due to either medications or malignancy) that are unrelated to PCD.

Roughly half of the patients (47%) underwent CT. Also, two thirds of the patients had an abnormal endoscopy, demonstrating that an unremarkable CT scan does not negate the need for a colonoscopy. Authors have reported undefined imaging and endoscopic findings indicative of colitis in other studies, as well.^18^ Only 7% of patients had PCD exclusively in the right colon. Therefore, sigmoidoscopy rather than the more invasive colonoscopy could be considered in patients with suspected colitis in whom colonoscopy may be risky. The presence of mucosal ulceration or nonulcerative inflammation according to endoscopy did not correlate with an increase in the administration of treatment of PCD, and neither did active or chronic inflammation according to histological examination. This is likely due to the fact that patients received treatment based on clinical PCD symptoms alone and not endoscopic findings. Again, the clinical picture is not predictive of endoscopic abnormalities, and both should be considered in evaluating a patient with suspected PCD.

Carboplatin recipients were the most likely to have PBT restarted after resolution of PCD and the least likely to have colitis complications. In the future, endoscopy in carboplatin recipients may be safer than that in patients receiving other platinum drugs. In contrast, oxaliplatin recipients had the highest prevalence of colitis complications but the fewest recurrent gastrointestinal symptoms. Clearly, initial clinical presentation does not correlate with future symptoms in patients with PCD. To minimize these side effects in the future, new classes of platinum agents are being developed (e.g., nedaplatin, lobaplatin, heptaplatin), but they have limited regional approval 19.

This study has some limitations. First, the retrospective design limited our ability to obtain detailed and accurate clinical information regarding medications used and their duration of use. Second, the absence of treatment standardization and uncertainty in PCD diagnosis led to arbitrary treatment approaches that may not have been based on evidence-based medicine. Third, including patients who underwent colonoscopic and histological evaluation could have precluded some cases of PCD. Fourth, despite being the largest study on this topic, the small sample size underpowers any meaningful analyses.

## Conclusions

PCD is rare, but in some patients, it can lead to severe complications requiring surgical intervention. The clinical presentation of PCD is nonspecific and can be confused with other etiologies of colitis. Still, endoscopic and histological findings can increase the degree of certainty of diagnosis and lead to prompt treatment to prevent adverse consequences. Resumption of PBT after PCD resolution results in mild gastrointestinal symptom recurrence in 25% of patients, which can be managed expectantly. Future studies investigating the appropriate treatment approaches for PCD are needed.

## Figures and Tables

**Table 1:** Patients' characteristics

Characteristic	Number of patients (%) (*n* = 86)
**Median age, years (IQR)**	57 (49-66)
**Male sex**	47 (55)
**Race**	
Non-Hispanic White	65 (76)
Black	11 (13)
Other	10 (12)
**NSAID use**	37 (43)
**Smoking**	48 (56)
**Comorbidities**	52 (60)
**Underlying IBD**	9 (10)
Active status	4 (5)
**Median ANC at time of PCD onset, k/mm^3^ (IQR)**	3.1 (1.4-4.2)
**Primary malignancy**	
Lung	18 (21)
Gastrointestinal	24 (28)
Gynecological	16 (19)
Genitourinary	9 (10)
Other	19 (22)
**Median duration of PBT, days (IQR)**	51 (21-114)
**Median time from PBT to PCD onset, days (IQR)**	66 (9-153)
**Platinum drug administered**	
Carboplatin	40 (47)
Cisplatin	27 (31)
Oxaliplatin	19 (22)
**Adjuvant chemotherapy**	
Taxane	24 (28)
FOLFOX	7 (8)
Combined regimen	28 (33)
Others	8 (9)
**PBT stopped due to PCD**	15 (17)
**Median duration of PCD symptoms, days (IQR)**	20 (7-56)
**Clinical PCD symptoms**	
Diarrhea	53 (62)
Abdominal pain	41 (48)
Blood or mucous in stool	37 (43)
Vomiting	17 (20)
Fever	5 (6)
**Peak diarrhea grade**	
1-2	41 (48)
3-4	13 (15)
**Peak colitis grade**	
1-2	68 (79)
3	11 (13)
**Median duration of PCD treatment, days (IQR)**	14 (7-29)
**Hospitalization**	42 (49)
**ICU admission**	8 (9)
**PCD treatment**	51 (59)
Immunosuppressants	18 (21)
Antibiotics	23 (27)
Antimotility agents	19 (22)
Intravenous fluids	44 (51)
**Colonic perforation requiring surgery**	6 (7)
**PBT restarted after PCD resolution**	37 (43)
**Recurrent PCD symptoms**	8 (9)
**CT evaluation**	40 (47)
Abnormal findings	27 (68)
**Colonoscopic findings**	
Ulcer	29 (34)
Inflammation	28 (33)
Normal	29 (34)
**Colonoscopic inflammatory pattern**	
Diffuse	20 (23)
Patchy	34 (40)
**Colitis location according to endoscopy**	
Entire colon	22 (26)
Left colon only	28 (33)
Right colon only	6 (7)
**Active inflammation according to histology**	65 (76)
**Chronic inflammation according to histology**	47 (55)
**Repeat endoscopy**	14 (16)
Endoscopic remission	5 (36)
Histological remission	3 (21)
**Overall mortality**	45 (52)

*NSAID* nonsteroidal anti-inflammatory drug, *IBD* inflammatory bowel disease, *ANC* absolute neutrophil count, *PCD* platinum-based therapy-related diarrhea and colitis. None of the mortality was related to GI toxicity.

**Table 2:** Patients' characteristics stratified according to platinum drug

Characteristic	Number of patients (%)			*P*
	Oxaliplatin (*n* = 19)	Carboplatin (*n* = 40)	Cisplatin (*n* = 27)	
**Median time from PBT to PCD onset, days (IQR)**	74 (36-148)	72 (12-166)	29 (6-137)	0.428
**Clinical PCD symptoms**				
Diarrhea	5 (26)	29 (73)	19 (70)	0.002
Abdominal pain	6 (32)	22 (55)	13 (48)	0.242
Blood or mucous in stool	4 (21)	20 (50)	13 (48)	0.090
Nausea and vomiting	4 (21)	7 (18)	6 (22)	0.882
Fever	0 (0)	1 (3)	4 (15)	0.051
**Median duration of PCD symptoms, days (IQR)**	7 (4-15)	30 (5-60)	25 (10-47)	0.182
**Hospitalization**	7 (37)	22 (55)	13 (48)	0.445
**ICU admission**	2 (11)	3 (8)	3 (11)	0.864
**Median duration of hospitalization, days (IQR)**	6 (2-9)	6 (3-15)	9 (6-14)	0.333
**CT abnormality^a^**	5 (26)	10 (25)	12 (44)	0.256
**Endoscopic presentation**				0.574
Ulcer	6 (32)	11 (28)	12 (44)	
Nonulcerative inflammation	5 (26)	15 (38)	8 (30)	
Normal	8 (42)	14 (35)	7 (26)	
**Colonoscopic colitis pattern^b^**				0.098
Diffuse	1 (5)	13 (33)	6 (22)	
Patchy	8 (42)	13 (33)	13 (48)	
**Active inflammation according to histology**	12 (63)	31 (78)	22 (81)	0.508
**Features of chronic inflammation**	6 (32)	24 (60)	17 (63)	0.100
**Colonic perforation requiring surgery**	3 (16)	0 (0)	3 (11)	0.050
**PCD treatment**	8 (42)	24 (60)	19 (70)	0.157
Immunosuppressants	1 (5)	10 (25)	7 (26)	0.163
Antibiotics	2 (11)	12 (30)	9 (33)	0.186
Antimotility agents	5 (26)	5 (13)	9 (33)	0.115
Intravenous fluids	7 (37)	22 (55)	15 (56)	0.393
**Repeat endoscopy**	7 (37)	5 (13)	2 (7)	0.020
**PBT restarted after PCD resolution**	5 (26)	23 (58)	9 (33)	0.037
**Recurrent PCD symptoms**	0 (0)	7 (18)	1 (4)	0.046

^a^Available for 27 patients. ^b^Available for 54 patients.

**Table 3:** Patients' characteristics stratified according to PCD treatment

Characteristic	Number of patients (%)		*P*
	Treatment^a^ (*n* = 51)	No treatment (*n* = 35)	
**Platinum drug**			0.157
Carboplatin	24 (47)	16 (46)	
Cisplatin	19 (37)	8 (23)	
Oxaliplatin	8 (16)	11 (31)	
**Clinical PCD symptoms**			
Diarrhea	41 (80)	12 (34)	<0.001
Abdominal pain	33 (65)	8 (23)	<0.001
Blood or mucous in stool	26 (51)	11 (31)	0.081
Vomiting	15 (29)	2 (6)	0.006
Fever	5 (10)	0 (0)	0.077
**Median duration of PCD symptoms, days (IQR)**	28 (9-56)	12 (3-53)	0.361
**Median duration of hospitalization, days (IQR)**	8 (3-14)	5 (3-9)	0.247
**CT abnormality^b^**	21 (41)	6 (17)	0.163
**Endoscopic presentation**			0.754
Ulcer	18 (35)	11 (31)	
Nonulcerative inflammation	15 (29)	13 (37)	
Normal	18 (35)	11 (31)	
**Active inflammation according to histology**	42 (82)	23 (66)	0.390
**Features of chronic inflammation**	31 (61)	16 (43)	0.367
**Colonic perforation requiring surgery**	4 (8)	2 (6)	1.000
**PBT restarted after PCD resolution**	22 (43)	15 (43)	1.000
**Repeat endoscopy**	7 (14)	7 (20)	0.555
**Recurrent PCD symptoms**	6 (12)	2 (6)	0.464

^a^Treatment of PCD included intravenous fluids, antibiotics, immunosuppressants, and antimotility agents. ^b^Available for 27 patients.
